# Quantifying the versatility of routinely measured prognostic factors

**DOI:** 10.1186/s41512-025-00206-7

**Published:** 2025-12-04

**Authors:** Hamish Innes, Philip J Johnson

**Affiliations:** 1 School of Health and Life Sciences, Glasgow Caledonian University, Glasgow, UK; 2 University of Nottingham, Lifespan and Population Health, Nottingham, UK; 3 Public Health Scotland, Blood Borne Virus and STI, Glasgow, UK; 4 Department of Molecular and Clinical Cancer Medicine, University of Liverpool, Liverpool, UK

**Keywords:** Prognosis, Clinical prediction models, Prognostic factors, Prediction, Personalised medicine, Algorithm

## Abstract

**Background:**

Some prognostic factors (PF) are “versatile” insofar as they predict diverse health outcomes (age is an exemplar par excellence). In this study, we sought to quantify the versatility of commonly measured PFs.

**Methods:**

Participants from the UKB (UK biobank) study were followed from enrolment until the date of outcome or censoring. Over 800 incident adverse outcomes were considered, based on a unique 3-digit ICD code (A00, A01, A02, etc.). Twenty-four routine PFs—including renal, liver function and blood count biomarkers—featured in this analysis. Cox regression was used to determine the association between each PF with time to each outcome event. The number of statistically significant associations, direction of the association (positive/negative) and the median log hazard ratio (LHR) were determined for each PF. Data were visualised using Volcano plots.

**Results:**

The analysis included up to 502,408 UKB participants followed for 12.4 years. PFs with the greatest number of statistically significant associations were age (563/836; median LHR: 0.47), waist-hip ratio (530/836; LHR: 0.37) and hand-grip strength (416/836; LHR: 0.27). Conversely, PFs with the lowest number of significant associations were diastolic blood pressure (138/835; LHR: 0.11) and total protein (138/835; LHR: 0.11). Positive correlation was observed between the number of events a PF was associated with and the average effect size for those associations.

**Conclusion:**

A wide spectrum exists between the most and least versatile PFs. In addition to age, waist-hip ratio and handgrip strength exhibit high versatility. Understanding PF versatility has implications for optimising the development/performance of prognostic models.

**Supplementary Information:**

The online version contains supplementary material available at 10.1186/s41512-025-00206-7.

## Highlights

- Prognostic factors (PFs) that are versatile are valuable from a health informatics perspective.
- This study evaluates the versatility of 24 routine prognostic factors (PFs) across >800 adverse events.
- A wide spectrum exists between the most and least versatile PFs.
- Age, waist-hip ratio, and handgrip strength were the most versatile PFs, associated with >50% of adverse health events.
- Versatile PFs not only predict more outcomes, but exhibit larger effect sizes for those outcomes too.
- Harnessing versatile PFs may help optimise performance of clinical prediction models.

**Supplementary Information:**

The online version contains supplementary material available at 10.1186/s41512-025-00206-7.

## Introduction

The function of a prognostic clinical prediction model (CPM) is to estimate a patient’s individualised risk of developing an adverse health outcome(s) over a future time horizon. In certain cases, this can help clinicians treat patients in a way that is more closely aligned to their needs (i.e. personalised medicine) [1]. 

Prognostic factors (PFs) represent the engine of a CPM. Indeed, it is through PFs that (prognostic) CPMs are able to discriminate between individuals who are likely and unlikely to develop an adverse health outcome in the future. Age is included as a PF in most CPMs [2–7]. In large part, this is because age associates with multiple diverse health outcomes. In this way, age can be considered a “versatile” PF. One might conceptualise a versatile PF in the following way: if one were to select 100 clinically distinct health outcomes, then a versatile PF would predict (i.e. associate with) a large proportion of these 100 outcomes. In contrast, a narrow/niche PF would associate with only a small proportion of these 100 events.

A “versatility” perspective is prominent in genetics. For example, after identifying genetic loci that associate with a target outcome, it is standard practice to perform a phenome-wide association scan (PheWAS) [8]. This entails testing that same genetic locus for association with a broader set of health outcomes. Genetic loci that associate with multiple health outcomes are said to be pleiotropic, which has implications for whether that locus would make a favourable drug target. Thus, a PheWAS can flag up genetic loci that are highly versatile/pleiotropic. An example of a versatile genetic variant is the APOE genotype, which simultaneously associates with the risk of Alzheimer’s disease, liver cancer, cirrhosis, obesity, ischaemic heart disease, type 2 diabetes, gallbladder disease and other outcomes [9]. Some of these associations lie in a favourable direction (e.g. liver disease) whereas others lie in an unfavourable direction (e.g. Alzheimers) [10, 11].

Outside of genetics, however, the concept (and implications) of versatility has not been well explored or discussed. In this study, we sought to quantify the versatility of common PFs measured in routine clinical practice and available in electronic health records.

## Methods

### UK Biobank

This study was carried out using the UK Biobank (UKB) resource (application #8764), a community cohort study of over half a million individuals from the UK [12]. Participants were interviewed from May 2006 to July 2010 at 22 UKB assessment centres located throughout the UK. All individuals aged 40–69 years and living within 25 miles of an assessment centre (approximately 9 million people in total) were sent an invitation letter for the study. During the interview, participants completed a comprehensive health questionnaire, a physical examination, and donated biological specimens. Follow-up data on subsequent health outcome events are supplied through record linkage to UK mortality, hospital admission, and cancer registries.

All laboratory tests were derived from blood specimens collected at UKB enrolment. The laboratory assays/methods used by UKB to generate these data have been described in detail [13, 14]. UKB has approval from the UK North West multi-centre Research Ethics Committee. Informed written consent was obtained from each participant.

### Prognostic factors

Twenty-four routine PFs were considered in this analysis:

- Renal tests: urea, creatinine, total protein.
- Cardiovascular: total cholesterol, HDL cholesterol, LDL cholesterol, systolic blood pressure, diastolic blood pressure
- Liver function tests: bilirubin, albumin, alanine aminotransferase, gamma-glutamyl transferase (GGT)
- Blood count: platelet count, red and white blood cell count, haemoglobin concentration
- BMI, waist-hip ratio, hand grip strength (Anthropometric measurements)
- Metabolic: glycated haemoglobin (HbA1c), glucose
- Socio-demographic factors: age, Townsend deprivation index
- Miscellaneous: C-reactive protein (CRP)

Selection of these PFs was guided by the reported frequency of use in UK primary care [15]. The specific UKB fields used to define each PF are provided in Table S1. N.B. all PFs considered above were continuous numeric variables.

### Study participants

This study included UK biobank participants who had not withdrawn from the study at the time our statistical analysis was commenced (*N* = 502,408).

Individuals with missing data for a given PF were excluded from the analysis for that specific PF, but not from the total study population. For example, if a participant was missing data on albumin level but not age, then they would contribute to the analysis of the latter but not the former.

For each PF, we also removed outlier data points using Tukey’s rule [16]. This defines a lower outlier as a data point less than Q1-3(IQR), where Q1 = 25th percentile, Q3 = 75th percentile, and IQR = interquartile range (i.e. the difference between Q1 and Q3). Conversely, an upper outlier is defined as a data point exceeding Q3 + 3(IQR). Alternative approaches to handling outlier data points were considered in sensitivity analyses. N.B. as missing data and outliers varied by PF, the final sample size also varied by PF. Outcome events:

We considered every possible 3-digit ICD code within an inpatient hospital admission as an adverse outcome event. In other words, outcome events range from ICD A00, A01, A02, A03, etc. through to N97, N98, and N99. All diagnostic positions within the hospital admission record were considered equally; for example, a participant was considered to have had an admission for A00, irrespective of whether that A00 code was recorded in the main discharge position or a supplementary position. Health outcomes (i.e. unique 3-digit ICD10 codes) with fewer than 20 events were excluded to ensure the PF-outcome association was measured precisely.

### Statistical analysis

Using Cox regression, we determined the association between each PF and time to first hospital admission for every adverse outcome.

As an example, 24 PFs, with 800 events per PF, would entail fitting 19,200 (24*800) separate models.

Our focus was to determine the association between the PF and *incident* disease. Thus, if an individual already had a hospital admission for a specific outcome (i.e. a given 3-digit ICD code) before enrolment, then they were excluded from the analysis of that event. This aligns with the standard approach to measuring disease incidence [17]. Also, if an individual did not have a hospital admission for this cause, their follow-up time was censored. The censoring date occurred at the earliest of either the patient’s death (if at all) or the date that our hospital admission data were complete until (28/2/2018 to 30/9/2021 depending on country of residence).

For Cox regression, all PFs were *Z* standardised to support the comparison of effect sizes between PFs. Statistical significance was defined as *p* < 0.05 with Bonferroni correction for multiple testing. For example, if 800 events were tested, the threshold for statistical significance would be < 0.000063 (i.e. 0.05/800). The total number of statistically significant associations for all health events was determined for each PF. For those statistically significant associations, we also determined the number of positive associations (i.e. log hazard ratio > 0) and negative (i.e. log hazard ratio < 0). Further, we extracted the absolute log hazard ratio (LHR) for each PF where a statistically significant association was observed.

Association was visualised using a volcano plot; this is essentially a scatter plot with log hazard ratio (*x*-axis) and *p*-value (*y*-axis), and where each data point corresponds to a specific adverse health outcome.

### Sensitivity analyses

Two key sensitivity analyses were performed. First, we re-ran our analysis using “blocks” of related ICD codes instead of unique 3-digit codes. Coding blocks include related 3-digit ICD codes and were defined using the WHO ICD-10 dictionary. For example, the “Influenza and pneumonia” block includes all ICD codes between J09 and J10. Table S2 outlines the coding blocks considered.

Secondly, we explored the impact of winsorising outlier values on versatility metrics as opposed to excluding outlier values.

## Results

### Cohort description

Table S3 indicates the number of participants included in each PF analysis, and after the exclusion of outliers and missing data.

The number of participants with outlier PF values was generally low. Exceptions were C-reactive protein, HbA1c, GGT, and glucose, where 12,000–24,000 data points were outliers.

In general, missing data was negligible for anthropometric and socio-demographic PFs (*n* < 5000), but more substantial for blood tests. In particular, missing data was greatest for glucose (*n* = 72,615) and total protein (*n* = 72,882).

Descriptive summary statistics for each PF are provided in Table 1. In total, over 800 adverse outcomes were assessed in this cohort. The median number of events per outcome was 556. The most frequent incident events were: I25 (Ischemic heart disease; *n* = 111,323); E78 (disorders of lipoprotein metabolism; *N* = 59,670); K57 (diversticular disease of intestine; *n* = 52,156); and K21 (gastro-oesophageal reflux disease) (Table S4).

**Table 1:**
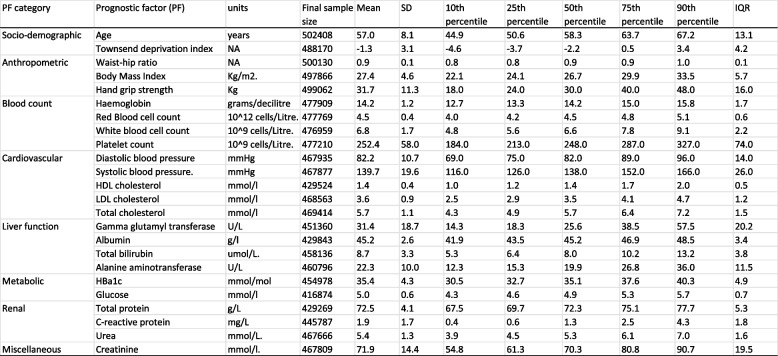
Summary and descriptive statistics of prognostic factors

The duration of follow-up exceeded 12 years across all event-specific analyses.

### Versatility

**Fig. 1:**
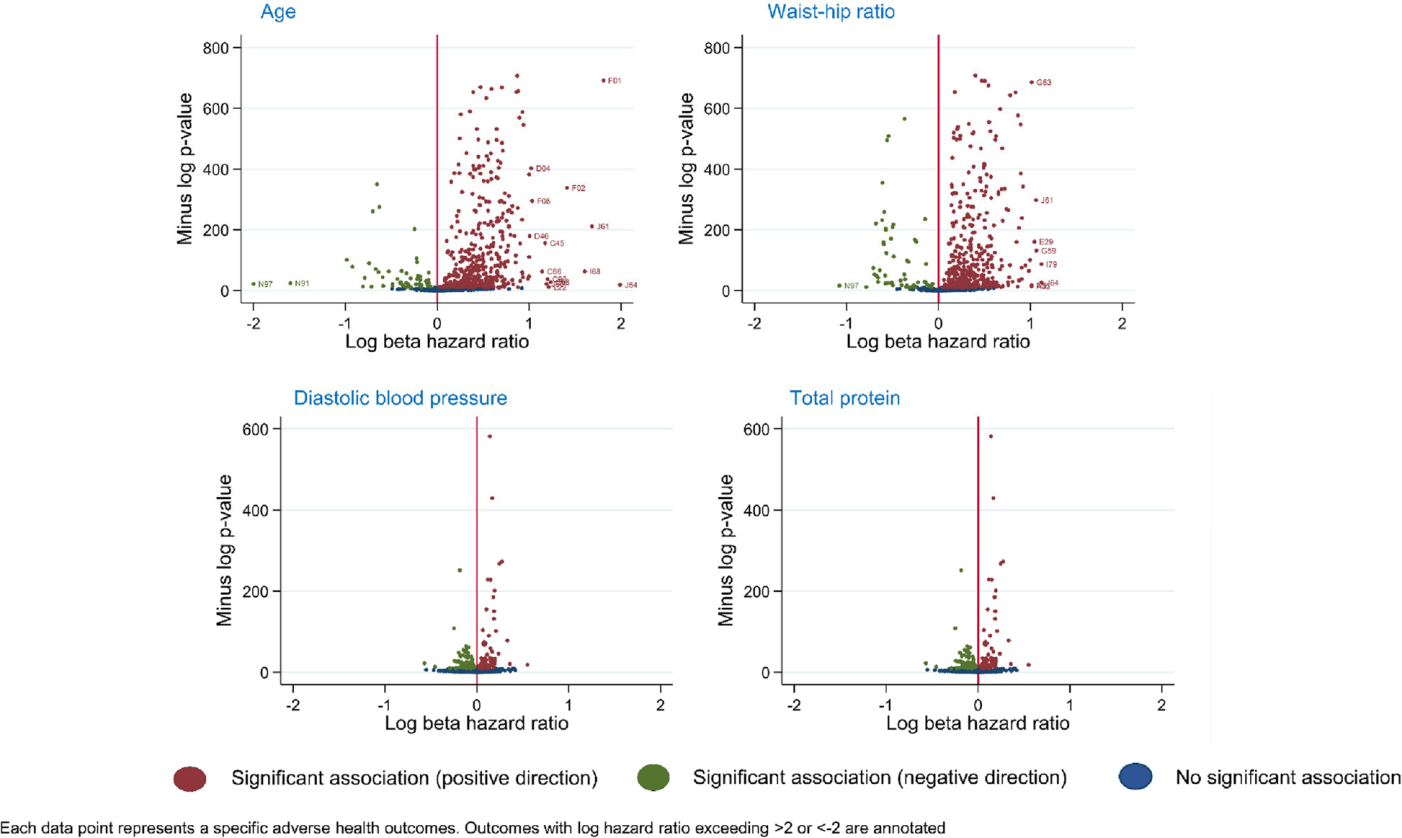
Volcano plots for most and least versatile prognostic factors

PFs with the greatest number of statistically significant associations were age (563/836; 67%); waist-hip ratio (550/836; 66%) handgrip strength (416/836; 50%) and C-reactive protein (408/836; 49%). (Table 2). Conversely, PFs with the least number of significant associations were diastolic blood pressure (138/835; 17%) and total protein (138/835; 17%). Thus, in this way, there was a stark gulf between the most and least versatile PFs (Fig. 1).

**Table 2:**
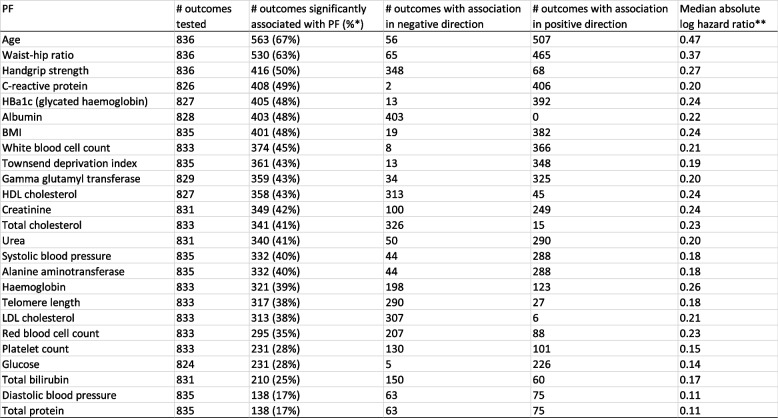
Prognostic factors (PFs) ordered by versatility

N.B. PFs arranged in descending order of the number of significance associations

*Percentage refers to the number of outcomes with a significant association as a fraction of the total number of outcomes tested

**Mean absolute log hazard ratio only includes events with a significant association. This, for age, 0.47 is the median absolute log hazard ratio among the 563 events that age was significantly associated with

**Fig. 2:**
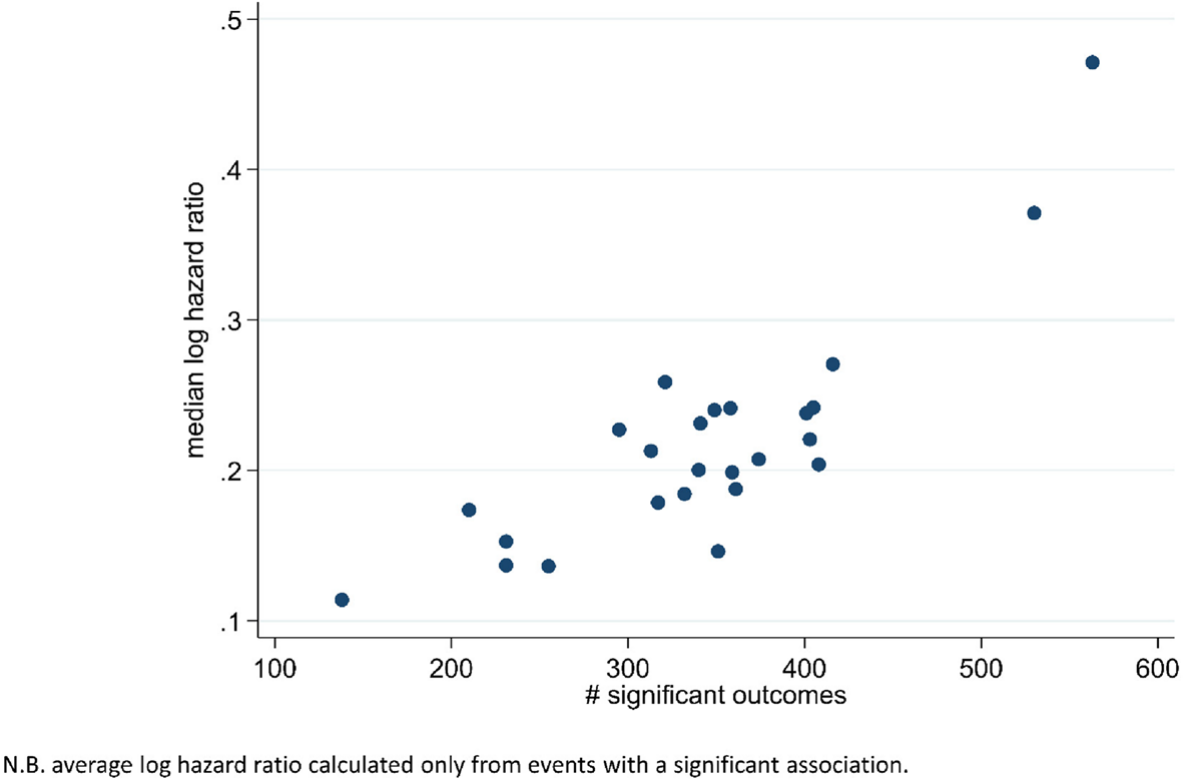
Correlation between the number of significant outcomes and median effect size. Each data point relates to a specific PF

Positive correlation was observed between the number of events a PF was associated with and the average effect size for those associations (Fig. 2). For example, age was associated with 563 events, of which the median log hazard ratio was 0.47. In contrast, diastolic blood pressure was associated with only 138 events, and the median log hazard ratio across those 138 associations was only 0.11.

**Fig. 3:**
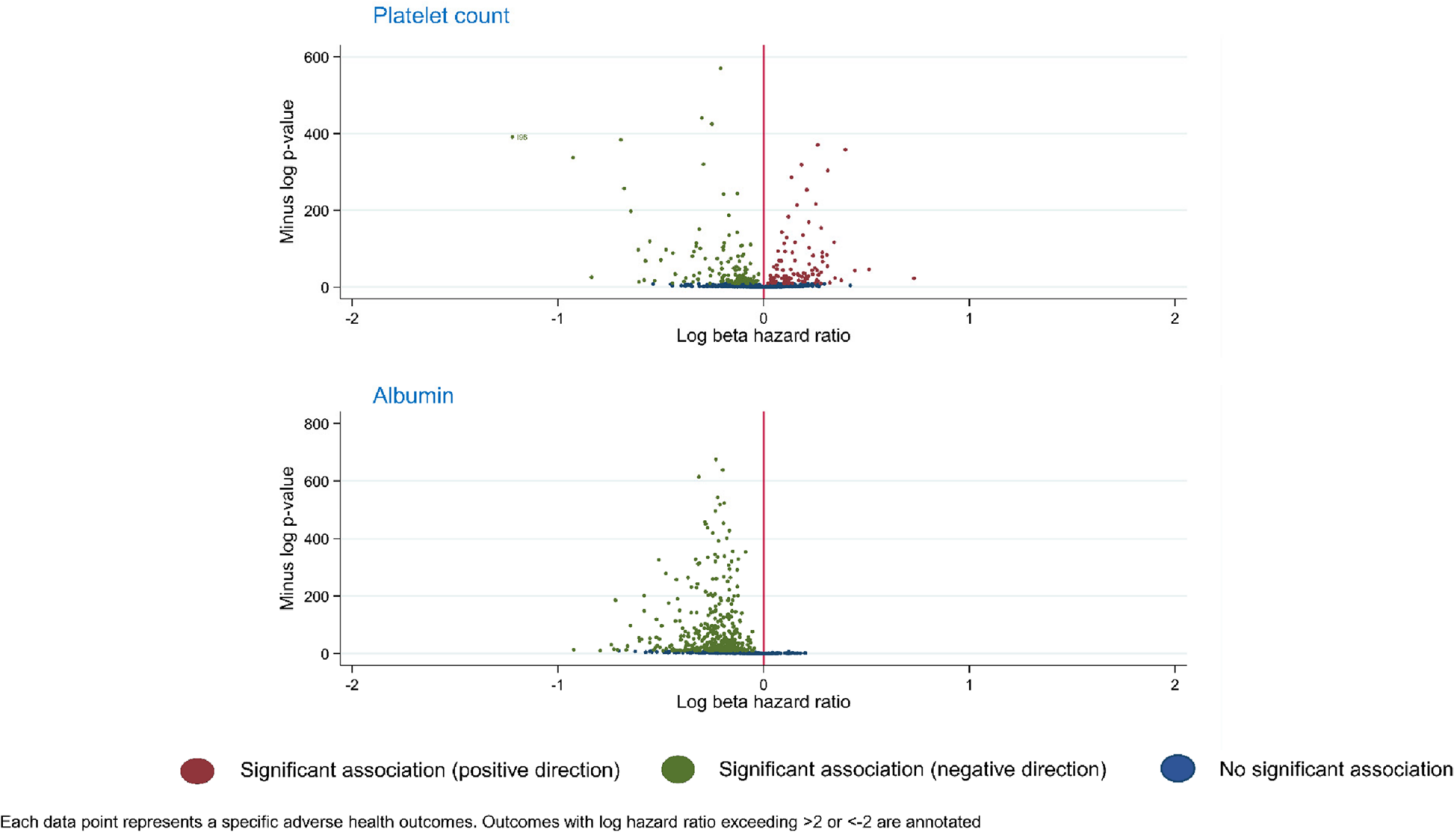
Contrast between prognostic factors with predominant effect in one direction versus both directions

PFs also varied in terms of the proportion of positive vs negative associations. For example, for albumin, all 412 significant associations were in the negative direction (i.e. higher albumin associated with lower risk of event). In contrast, for platelet count, the number of associations in the negative (*n* = 121) and positive (*n* = 111) direction was roughly comparable (Fig. 3).

### Sensitivity analyses

**Fig. 4:**
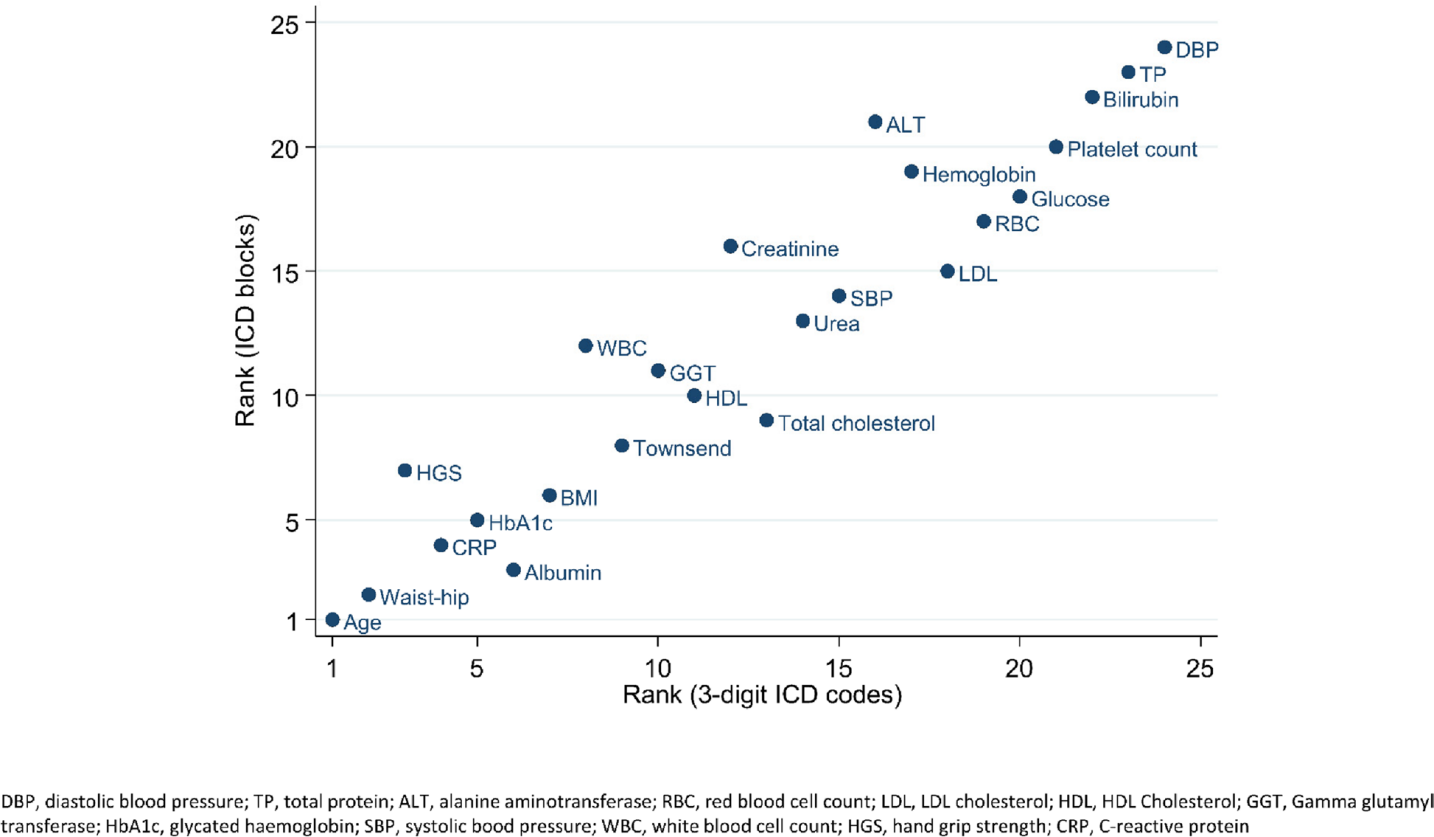
Correlation between versatility ranking via 3-digit ICD code vs. ICD code blocks

When using broader ICD coding blocks instead of specific 3-digit codes, the PF versatility ranking was not materially altered (see Fig. 4; Table S5). Winsoring outlier values (as opposed to excluding outlier values) increased the number of significant associations, but only modestly. Conversely, ignoring outlier values constrained PF effect sizes considerably (Fig. S1–3; Table S6).

## Discussion

This study is the first to describe the versatility of PFs used in routine practice. Our results indicate a wide spectrum between the most and least versatile PFs. Age in particular emerges as singular, but we spotlight additional PFs that are highly versatile too (namely, waist hip ratio, hand grip strength and C-reactive protein). Indeed, waist-hip ratio in particular was only slightly behind age in terms of the number of significant associations and median effect size.

Equally, our results draw attention to routine PFs whose versatility is relatively low (total protein, systolic blood pressure). The low versatility of blood pressure (both systolic and diastolic) relative to other PFs is arguably surprising given its longstanding use as a first-line test in general practice. Similarly, BMI exhibits inferior versatility to waist–hip circumference, despite the latter being more widely used.

Understanding PF versatility is important for several reasons. First, we show that the hallmark of a “versatile” PF is not only that it associates with/predicts more outcomes, but that it exhibits larger effect sizes for these outcomes too. This underscores the importance of harnessing versatile PFs to optimise CPM performance.

Second, deciding which PFs to collect data for and/or include in a given CPM is a crucial decision point that can have real impact on a CPM’s discriminative performance. Current guidelines recommend that PFs should be selected through subject-specific knowledge [18, 19], for example based on a systematic review of the literature [20]. However, the scientific literature can be incomplete (particularly for rarer outcomes) meaning that there is real potential for relevant PFs to be omitted. As a result, the predictive power of a CPM is constrained before the model has even been fitted. A possible refinement of current practice, therefore, might be to begin with a core set of highly versatile PFs to which niche PFs supported by subject knowledge could then be added. This approach may augment model performance. In fact, one can argue that this strategy is already (tacitly) being practiced with respect to age, which is included in almost all CPMs. Our study suggests that this default inclusion approach currently applied to age might be considered for other PFs as well.

PF versatility also intersects another important weakness of existing CPMs: namely, that whilst individuals with chronic diseases are susceptible to multiple adverse events, current CPMs overwhelmingly predict single disease outcomes alone [2–7]. With multi-morbidity rising [21], “overarching” CPMs that predict multiple outcomes simultaneously will be increasingly needed to foster holistic and proportionate decision making [22]. This is likely to be the direction of travel for CPMs over the next ten years, with such models already emerging [23]. To predict multiple outcomes with sufficient accuracy, however, we suggest that such CPMs will need to be underpinned by versatile PFs. Thus, understanding PF versatility will become increasingly salient in this sphere and indeed may influence what PFs are measured/collected by health care systems. We believe our study is relevant to this trajectory.

Finally, it is anticipated that new biomarkers, based on increasingly sophisticated (and more costly) technologies, will become available in the years ahead. For example, epigenetic biomarkers attempt to capture an individual’s biological age, and thus surpass the predictive value of chronological age [24]. Polygenic risk scores based on germline DNA are also being increasingly utilised and explored [25]. The analysis adopted herein may offer a useful (and impartial) framework for evaluating the global value of such new biomarker(s) relative to existing alternatives.

A key strength of this study is the exploration of an emerging topic that is not yet well understood or characterised. We also leverage the powerful UK Biobank resource, an ideal cohort to study PF versatility due to its large sample size, breadth of prognostic factors, and maturity vis-à-vis a long duration of follow-up. Our analysis does have limitations; however, that merit discussion.

PF versatility has not been widely discussed as a concept and there may be different legitimate views about what this means and how it should be defined. Our working definition—i.e. the ability of PF to predict/associate with multiple clinically distinct events—is admittedly loose and may not capture all nuances of the versatility concept. Indeed, there is an element of subjectivity about deciding whether two events are clinically distinct or not. Further, “clinically distinct” does not necessarily mean “biologically distinct”, which others may see as a stronger test of a prognostic factor’s versatility. For example, liver disease, type 2 diabetes and cardiovascular disease are clinically distinct (insofar as they are managed by different specialities) yet they share important biological pathways/mechanisms. However, one could argue that such biological overlap is the norm rather than the exception.

Arguably, another relevant aspect of versatility is the ability of a PF to produce diverse effect sizes across a set of outcomes. This dimension was best demonstrated by platelet count in this study, which exhibited a range of strong positive and negative associations (Fig. 3). Also, as an alternative to quantifying the number of events a PF associates with, one might choose to express versatility in terms of the number of associated events weighted by a measure of importance (i.e. perhaps the outcome event’s incidence rate or clinical severity).

Versatility might alternatively be framed as a lack of specificity vis-à-vis the association between a specific PF and outcome. Historically, the lack of specificity has been perceived as a negative quality in disciplines such as epidemiology, e.g. undermining the case for a causal relationship, as per the Bradford Hill criteria. In the era of big data, however, PFs that associate with numerous outcomes but lack specificity to any particular one could be useful and may become sought after.

Although we have focused on the versatility of individuals’ PFs, this concept presumably extends to multivariate risk scores too (i.e. derivatives of PFs). The ALBI score, originally developed to measure liver function in patients with liver cancer, is one potential example that is associated with many “off target” events [26]. A consensus is needed to formalise the definition of versatility to support future studies exploring this concept.

Our study has several methodological limitations. As this analysis was computationally demanding (fitting 19,200 separate models for our base-case analysis alone), several of these limitations reflect constraints in the computational resources available to us.

First, we only considered continuous variables. Thus, categorical factors such as gender and ethnicity were not included. This is because statistical power to detect associations is, ceteris paribus, weaker for categorical variables versus continuous variables— hence, we felt that metrics such as the number of events with a significant association and median effect size may not be comparable between these different variable classes.

We also did not take competing risk events into account, mainly due to the greater computational demands required to fit a Fine-Gray model with a large sample size relative to a Cox model.

Further, given the large number of models fitted, we were unable to check the proportional hazards assumption. Studies show that where this assumption is violated, hazard ratios are influenced by the censoring distribution and thus may not be reproducible between studies [27].

In addition, we did not consider the potential for non-linear associations between PFs and adverse health outcomes. We also excluded individuals with missing data and did not adopt multiple imputation to minimise bias, mainly because this would have required us to replicate our analysis in each imputation data (*n* = 10 at minimum), which would not have been computationally feasible for us. However, with the exception of total protein and glucose, missing PF data was relatively infrequent.

Another caveat is that PFs for which missing data were infrequent (i.e. age) were probably better powered to achieve statistically significant associations versus PFs where missing data was more frequent (e.g. total protein). On the whole, however, differences in sample size between PFs were modest and are unlikely to have impacted the versatility spectrum reported.

A strength of this analysis is that we adopted a wide scope in terms of reporting > 800 health outcomes defined via 3-digit ICD codes. Many of these events are not clinically distinct; however, for example, a patient admitted to hospital for K70 (alcohol liver disease) is also more likely to be admitted to hospital for K74 (fibrosis and cirrhosis of the liver). Indeed, these two codes are often recorded simultaneously within the same admission. Thus, a PF associated with K70 will also tend to be associated with K74. These thoughts motivated us to perform a sensitivity analysis where adverse outcomes were defined on the basis of broader coding blocks (e.g. K70-K77; disease of the liver) where greater independence between events can be assumed. It is noteworthy that in this analysis, we saw a very similar ranking in terms of the most and least versatile PFs. Finally, we did not consider the versatility of existing CPMs such as QRisk [4]; this is another related area that could be explored in future work.

In summary, this study describes the versatility of routine PFs for the first time. A versatility perspective may help to optimise the development of CPMs, particularly for pan-outcome models. More research is needed to explore this topic further.

## Supplementary Information

Supplementary Material 1: Figure S1. Alternative approaches to handling outliers datapoints: C-reactive protein.

Supplementary Material 2: Figure S2. Alternative approaches to handling outliers datapoints: Gamma glutamyl transferase.

Supplementary Material 3: Figure S3. Alternative approaches to handling outliers datapoints: Gamma glutamyl transferase.

Supplementary Material 4: Table S1. derivation of prognostic factors.

Supplementary Material 5: Table S3: Derivation of final sample size.

Supplementary Material 6: Table S4. Ten most frequent outcomes.

Supplementary Material 7: Table S5. Prognostic factorsordered by versatility. Outcomes defined by ICD coding blocks as opposed to 3-digit ICD codes.

Supplementary Material 8: Table S6. Impact on versatility metrics of alternative approaches to handling outlier data points.

## Data Availability

This research has been conducted using the United Kingdom (UK) Biobank Resource (application number: 8764). Data from the UK biobank are accessible to bona-fide researchers. For information about the application procedure, please see: [https://www.ukbiobank.ac.uk/enable-your-research/apply-for-access].
