# Supplementary figures and images for: Quantifying the versatility of routinely measured prognostic factors
Source: Diagn Progn Res. 2025 Dec 4;9:25. doi: 10.1186/s41512-025-00206-7 (PMC12676796; doi:10.1186/s41512-025-00206-7)

**
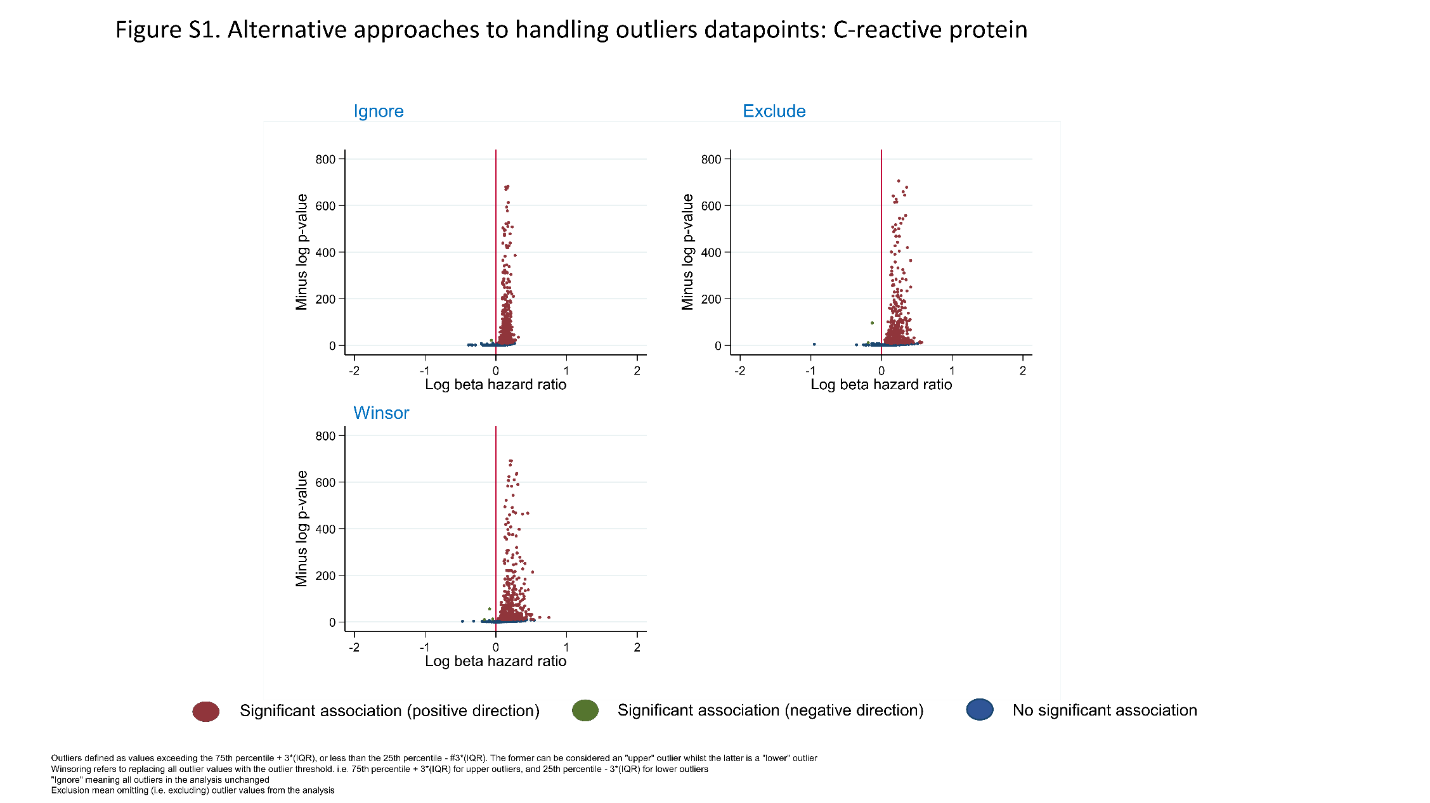
**

Supplement: Supplementary file 1 — Supplementary Material 1: Figure S1. Alternative approaches to handling outliers datapoints: C-reactive protein. [file 41512_2025_206_MOESM1_ESM.docx]

**
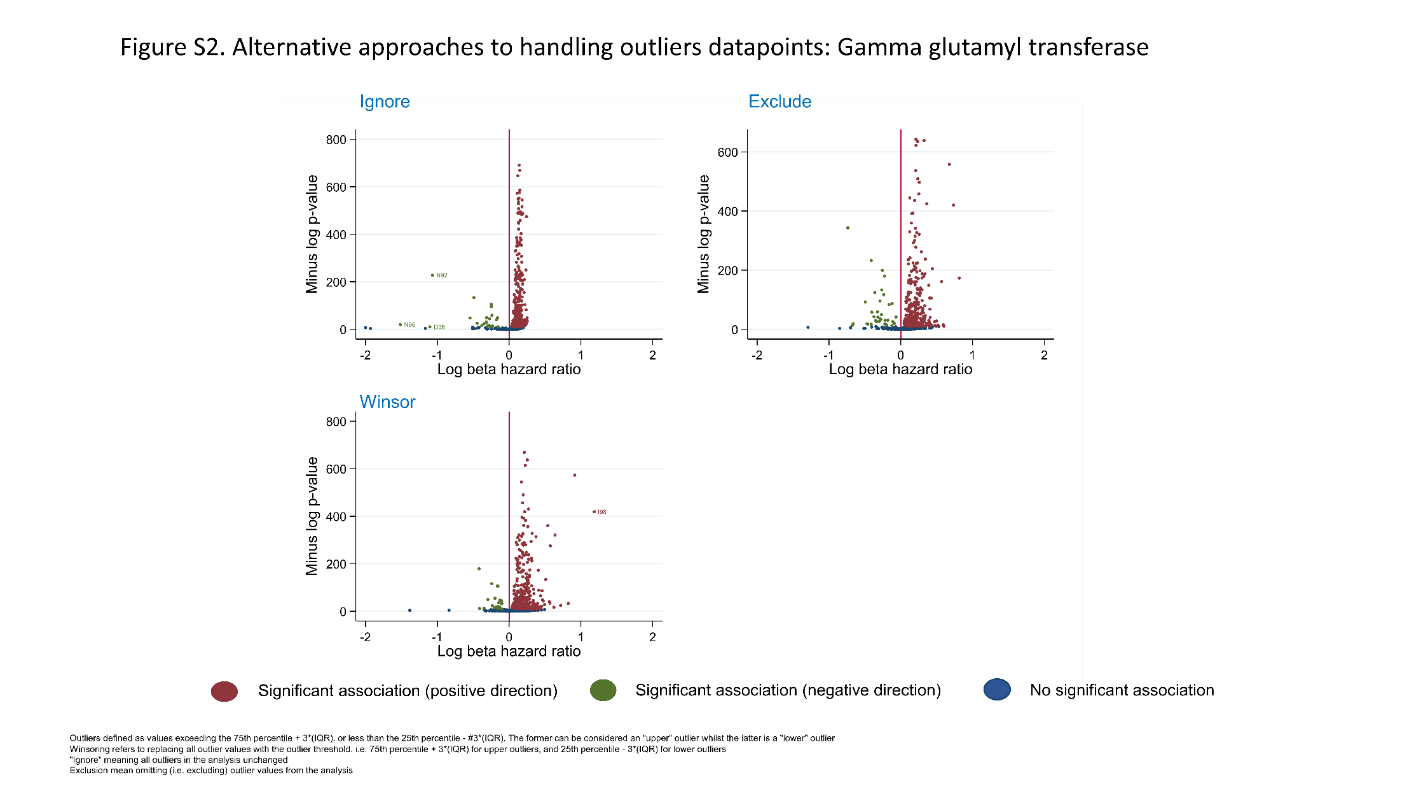
**

Supplement: Supplementary file 2 — Supplementary Material 2: Figure S2. Alternative approaches to handling outliers datapoints: Gamma glutamyl transferase. [file 41512_2025_206_MOESM2_ESM.docx]

**
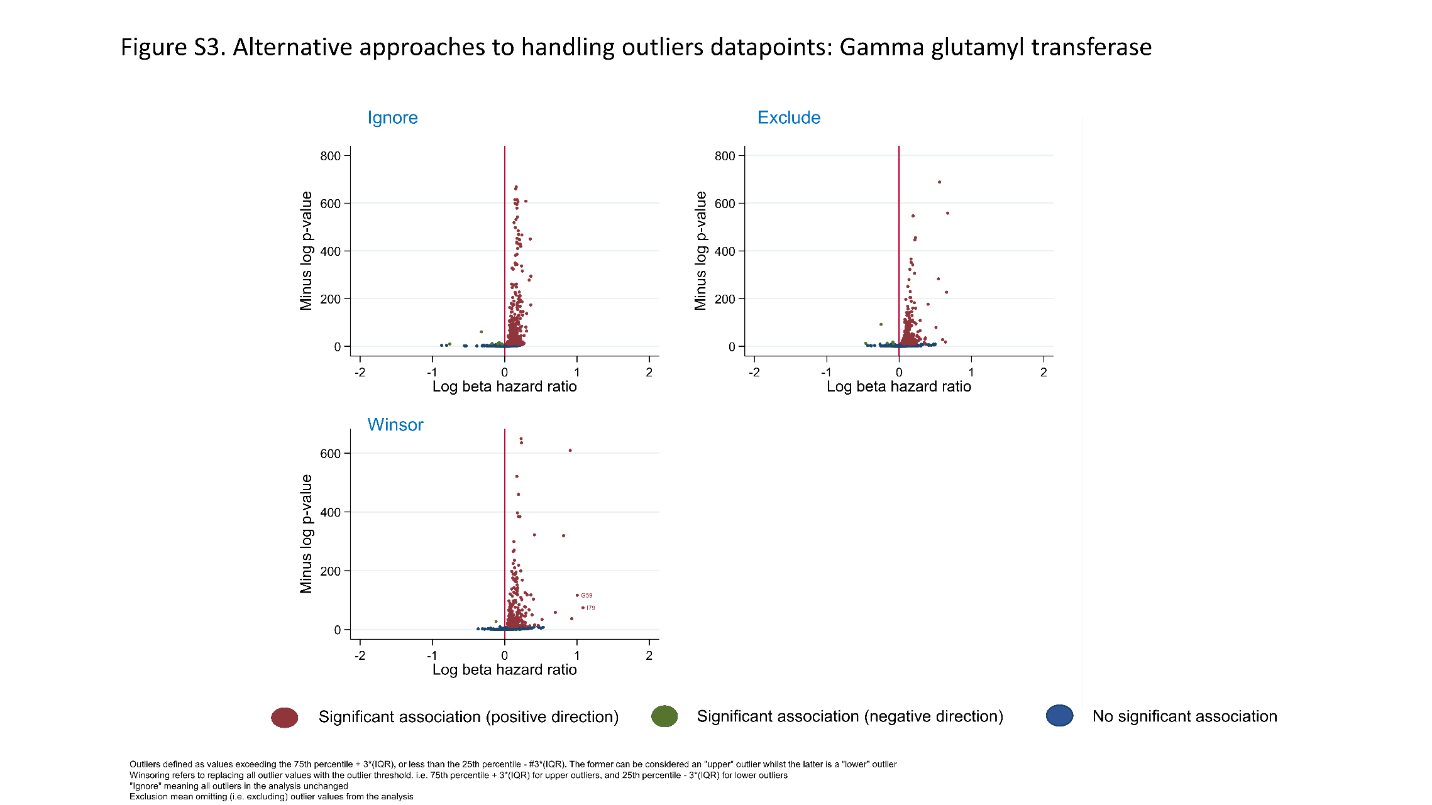
**

Supplement: Supplementary file 3 — Supplementary Material 3: Figure S3. Alternative approaches to handling outliers datapoints: Gamma glutamyl transferase. [file 41512_2025_206_MOESM3_ESM.docx]
