# Supplementary material for: Quantifying the versatility of routinely measured prognostic factors
Source: Diagn Progn Res. 2025 Dec 4;9:25. doi: 10.1186/s41512-025-00206-7 (PMC12676796; doi:10.1186/s41512-025-00206-7)
Supplement: Supplementary file 2 [file 41512_2025_206_MOESM4_ESM.docx]

Table S1. derivation of prognostic factors (PFs)

PF category	PF	UK field number	UKB Showcase link
Socio-demographic	Age	21022	https://biobank.ndph.ox.ac.uk/showcase/field.cgi?id=21022
	Townsend deprivation index	22189	https://biobank.ndph.ox.ac.uk/showcase/field.cgi?id=22189
Anthropometric	Waist-hip ratio	48 and 49	**48:** https://biobank.ndph.ox.ac.uk/showcase/field.cgi?id=48 ; **49:** https://biobank.ndph.ox.ac.uk/showcase/field.cgi?id=49
	Body Mass Index	21001	https://biobank.ndph.ox.ac.uk/showcase/field.cgi?id=21001
	Hand grip strength	46 and 47*	**46:** https://biobank.ndph.ox.ac.uk/showcase/field.cgi?id=46; **47:** https://biobank.ndph.ox.ac.uk/showcase/field.cgi?id=47
Blood count	Haemoglobin	30020	https://biobank.ndph.ox.ac.uk/showcase/field.cgi?id=30020
	Red Blood cell count	30170	https://biobank.ndph.ox.ac.uk/showcase/field.cgi?id=30170
	White blood cell count	30000	https://biobank.ndph.ox.ac.uk/showcase/field.cgi?id=30000
	Platelet count	30080	https://biobank.ndph.ox.ac.uk/showcase/field.cgi?id=30080
Cardiovascular	Diastolic blood pressure	4079	https://biobank.ndph.ox.ac.uk/showcase/field.cgi?id=4079
	Systolic blood pressure.	4080	https://biobank.ndph.ox.ac.uk/showcase/field.cgi?id=4080
	HDL cholesterol	23406	https://biobank.ndph.ox.ac.uk/showcase/field.cgi?id=23406
	LDL cholesterol	30780	https://biobank.ndph.ox.ac.uk/showcase/field.cgi?id=30780
	Total cholesterol	30690	https://biobank.ndph.ox.ac.uk/showcase/field.cgi?id=30690
Liver function	Gamma glutamyl transferase	30730	https://biobank.ndph.ox.ac.uk/showcase/field.cgi?id=30730
	Albumin	30600	https://biobank.ndph.ox.ac.uk/showcase/field.cgi?id=30600
	Total bilirubin	30840	https://biobank.ndph.ox.ac.uk/showcase/field.cgi?id=30840
	Alanine aminotransferase	30620	https://biobank.ndph.ox.ac.uk/showcase/field.cgi?id=30620
Metabolic	HBa1c	30750	https://biobank.ndph.ox.ac.uk/showcase/field.cgi?id=30750
	Glucose	30740	https://biobank.ndph.ox.ac.uk/showcase/field.cgi?id=30740
Renal	Total protein	30860	https://biobank.ndph.ox.ac.uk/showcase/field.cgi?id=30860
	C-reactive protein	30710	https://biobank.ndph.ox.ac.uk/showcase/field.cgi?id=30710
	Urea	30670	https://biobank.ndph.ox.ac.uk/showcase/field.cgi?id=30670
Miscellaneous	Creatinine	30700	https://biobank.ndph.ox.ac.uk/showcase/field.cgi?id=30700

*For this analysis, grip strength values were defined as average of left and right hand grip strength
