# Supplementary material for: Quantifying the versatility of routinely measured prognostic factors
Source: Diagn Progn Res. 2025 Dec 4;9:25. doi: 10.1186/s41512-025-00206-7 (PMC12676796; doi:10.1186/s41512-025-00206-7)
Supplement: Supplementary file 3 [file 41512_2025_206_MOESM5_ESM.docx]

Table S3: Derivation of final sample size.

PF category	Prognostic factor (PF)	Initial sample size	# missing data	# exceeding upper outlier	# exceeding lower outlier	Final sample size
Socio-demographic	Age	502408	0	0	0	502408
	Townsend deprivation index		14238	0	0	488170
Anthropometric	Waist-hip ratio		2265	12	1	500130
	Body Mass Index		3104	1438	0	497866
	Hand grip strength		3343	3	0	499062
Blood count	Haemoglobin		24326	7	166	477909
	Red Blood cell count		24326	134	179	477769
	White blood cell count		24331	1118	0	476959
	Platelet count		24329	869	0	477210
Cardiovascular	Diastolic blood pressure		34424	48	1	467935
	Systolic blood pressure.		34437	94	0	467877
	HDL cholesterol		72615	269	0	429524
	LDL cholesterol		33789	56	0	468563
	Total cholesterol		32906	88	0	469414
Liver function	Gamma glutamyl transferase		33153	17895	0	451360
	Albumin		72411	20	134	429843
	Total bilirubin		34935	9337	0	458136
	Alanine aminotransferase		33094	8518	0	460796
Metabolic	HBa1c		35994	11354	82	454978
	Glucose		72919	12499	116	416874
Renal	Total protein		72882	225	32	429269
	C-reactive protein		33927	22694	0	445787
	Urea		33224	1518	0	467666
Miscellaneous	Creatinine		33138	1461	0	467809
