# Supplementary material for: Quantifying the versatility of routinely measured prognostic factors
Source: Diagn Progn Res. 2025 Dec 4;9:25. doi: 10.1186/s41512-025-00206-7 (PMC12676796; doi:10.1186/s41512-025-00206-7)
Supplement: Supplementary file 4 [file 41512_2025_206_MOESM6_ESM.docx]

Table S4. Ten most frequent outcomes

ICD code	Description	number of incident events
I10	Essential (primary) hypertension	111323
E78	Disorders of lipoprotein metabolism and other lipidaemias	59670
K57	Diverticular disease of intestine	52156
K21	Gastro-oesophageal reflux disease	41739
M19	Other arthrosis	38622
K29	Gastritis and duodenitis	37998
K44	Diaphragmatic hernia	36659
H26	Other cataract	33966
I25	Chronic ischaemic heart disease	33605
E66	Obesity	32211

The specific number of incident events will vary by prognostic factor (i.e. due to variable missing data and outliers). The numbers in this table relate to the analysis with age as a prognostic factor.
