# Supplementary material for: Quantifying the versatility of routinely measured prognostic factors
Source: Diagn Progn Res. 2025 Dec 4;9:25. doi: 10.1186/s41512-025-00206-7 (PMC12676796; doi:10.1186/s41512-025-00206-7)
Supplement: Supplementary file 5 [file 41512_2025_206_MOESM7_ESM.docx]

Table S5. Prognostic factors (PFs) ordered by versatility. Outcomes defined by ICD coding blocks as opposed to 3-digit ICD codes

PF	# outcomes tested	# outcomes significantly associated with PF (%*)	# outcomes with association in negative direction	# outcomes with association in positive direction	Median absolute log hazard ratio**
Age	134	111 (83%)	9	102	0.39
Waist-hip ratio	134	108 (81%)	9	99	0.26
Albumin	133	95 (71%)	95	0	0.17
C-reactive protein	134	93 (70%)	1	92	0.15
HBa1c	134	90 (67%)	1	89	0.17
BMI	134	88 (66%)	5	83	0.17
Handgrip strength	134	86 (64%)	72	14	0.21
Townsend deprivation index	134	82 (61%)	9	73	0.14
Gamma glutamyl transferase	133	81 (60%)	5	76	0.15
Total cholesterol	134	81 (60%)	77	4	0.19
HDL Cholesterol	133	81 (60%)	73	8	0.19
White blood cell count	134	80 (60%)	0	80	0.16
Urea	134	80 (60%)	9	71	0.17
Telomere length	134	77 (57%)	70	7	0.15
Systolic blood pressure	134	75 (56%)	6	69	0.15
LDL Cholesterol	134	73 (54%)	69	4	0.16
Creatinine	134	69 (51%)	17	52	0.18
Red blood cell count	134	65 (49%)	48	17	0.14
Glucose	133	61 (46%)	1	60	0.11
Haemoglobin	134	60 (45%)	38	22	0.17
Platelet cout	134	58 (43%)	32	26	0.11
Alanine aminotransferase	133	57 (43%)	14	43	0.10
Bilirubin	133	48 (36%)	35	13	0.12
Total protein	133	40 (30%)	36	4	0.06
Diastolic blood pressure	134	37 (28%)	16	21	0.08

N.B PFs arranged in descending order of # significant associations.

* percentage refers to the number of outcomes with a significant association as a fraction of the total number of outcomes tested

**mean absolute log hazard ratio only includes events with a significant association. Thus, for age, 0.47 is the median absolute log hazard ratio among the 563 events that age was significantly associated with.
