# Supplementary material for: Quantifying the versatility of routinely measured prognostic factors
Source: Diagn Progn Res. 2025 Dec 4;9:25. doi: 10.1186/s41512-025-00206-7 (PMC12676796; doi:10.1186/s41512-025-00206-7)
Supplement: Supplementary file 6 [file 41512_2025_206_MOESM8_ESM.docx]

Table S6. Impact on versatility metrics of alternative approaches to handling outlier data points

PF	Approach to handling outliers	# outcomes tested	# outcomes significantly associated with PF (%)	# outcomes with association in negative direction	# outcomes with association in positive direction	Median absolute log hazard ratio
C-reactive protein	Winsoring	836	427 (51%)	3	424	0.21
	Ignore	831	418 (50%)	1	417	0.15
	Exclusion (basecase)	826	408 (49%)	2	406	0.20
GGT	Ignore	833	410 (49%)	23	387	0.13
	Winsoring	836	370 (44%)	23	347	0.19
	Exclusion (basecase)	829	359 (43%)	34	325	0.20
Glucose	Ignore	827	351 (42%)	8	343	0.15
	Winsoring	836	255 (31%)	1	254	0.14
	Exclusion (basecase)	824	231 (28%)	5	226	0.14

N.B. this table focuses on C-reactive protein, GGT and glucose, as these PFs had the highest level of outliers

Outliers defined as values exceeding the 75th percentile + 3*(IQR), or less than the 25th percentile - #3*(IQR). The former can be considered an "upper" outlier whilst the latter is a "lower" outlier

Winsoring refers to replacing all outlier values with the outlier threshold. i.e. 75th percentile + 3*(IQR) for upper outliers, and 25th percentile - 3*(IQR) for lower outliers

"Ignore" meaning all outliers in the analysis unchanged

Exclusion mean omitting (i.e. excluding) outlier values from the analysis
